# Editorial

**Published:** 1839-09-14

**Authors:** 


### THE MEDICAL EXAMINER.

### PHILADELPHIA, SEPTEMBER 14, 1839.

In one of our preceding numbers, we published a letter from a Parisian correspondent, which contained an interesting summary of a lecture of Dr. Dubois, (of Amiens,) on contagion and infection. The action of government in France is proverbially slow, and ancient customs are very reluctantly infringed; hence, the system of quarantine regulations subsists there nearly in its original form. A very slight modification in the rigour of these laws has lately been made, which diminishes the duration of quarantine for vessels arriving from the West Indies, or from American ports. No indulgence has yet been granted to vessels from the Levant, which are still obliged to undergo a rigorous quarantine in all the ports of the South of France.

The quarantine system is by some regarded as useless and injurious. This belief is founded on the difficulty of enforcing absolute exclusion of individuals who may labour under a contagious or infectious disorder, and on the evident fact that few diseases which are the subjects of quarantine regulations, are really susceptible of communication from a patient to healthy individuals. Nevertheless, it is undoubted that the regulations of a well-ordered quarantine are most useful in preventing large numbers of diseased individuals, and the clothing and bedding which they may bring with them, from entering those cities which would favour the propagation of disease.

The diseases which are susceptible of contagion by the direct contact of morbid matter, generated in the human body, are of course very few in number; but those which possess the property of infecting healthy individuals, who are exposed to the emanations of a large number of diseased persons, are by no means rare, although they do not seem to possess the property of infection under all circumstances. They must be well characterized and severe forms of the disease, and not the slighter varieties of the affection. For example, typhus fever is most highly infectious, when it is of a malignant character; and the records of medicine, from the time of the black assizes down to the typhus of 1813, are full of cases which illustrate this property of the disease. But when, from the season of the year, or some other cause, the disease is a mild one, it rarely extends beyond a very small number of individuals. At Philadelphia, we had two very dissimilar but partial epidemics of typhus fever, in the years 1836 and '37. The former was severe, and the cases were extremely grave; the latter was extremely mild. In the severe epidemic, a large number of individuals exposed to the exhalations from the patients, were taken down with the fever, but in the milder epidemic those in attendance upon the sick were exposed to the disease with perfect impunity. In epidemics of dysentery, we observe the same infectious character of the disease; the emanations from the persons and from the alvine discharges of patients, very often communicate the disorder, while sporadic and mild cases are perfectly free from such property. Typhoid fever is very rarely infectious, but in certain epidemics there is very strong ground for believing that the infectious quality is acquired.

Diseases which are avowedly infectious vary as to the intensity in which this quality is possessed, and follow the general rule that the more severe the form of the epidemic, the more apt is it to assume a highly infectious form.

It is somewhat difficult to say in what the infectious principle resides. Probably it depends upon a miasm, or gas, generated in the body of the patient. This would seem to be exhaled both from the mucous membranes and the surface of the body, particularly from the mucous surfaces. The peculiar smell characteristic of most diseases which become infectious, is most highly developed where this quality exists in the greatest intensity.

It will be seen that much of the confusion of ideas relative to infection and contagion, depends upon the want of definite notions upon the subject, and, perhaps, in a still greater degree, upon the varying intensity of the infectious property. The subject is one which often becomes a question of grave consideration for the physician, and therefore cannot be too carefully sifted, and cleared from all the extraneous matter which renders it obscure.

